# Including traffic jam avoidance in an agent-based network model

**DOI:** 10.1186/s40649-018-0053-y

**Published:** 2018-05-14

**Authors:** Christian Hofer, Georg Jäger, Manfred Füllsack

**Affiliations:** Institute of Systems Sciences, Innovation and Sustainability Research, University of Graz, Merangasse 18, 8010 Graz, Austria

**Keywords:** Agent-based model, Spatial networks, Traffic simulation, Origin–destination data, Congestion analysis

## Abstract

**Background:**

Understanding traffic is an important challenge in different scientific fields. While there are many approaches to constructing traffic models, most of them rely on origin–destination data and have difficulties when phenomena should be investigated that have an effect on the origin–destination matrix.

**Methods:**

A macroscopic traffic model is introduced that is novel in the sense that no origin–destination data are required as an input. This information is generated from mobility behavior data using a hybrid approach between agent-based modeling to find the origin and destination points of each vehicle and network techniques to find efficiently the routes most likely used to connect those points. The simulated road utilization and resulting congestion is compared to traffic data to quantitatively evaluate the results. Traffic jam avoidance behavior is included in the model in several variants, which are then all evaluated quantitatively.

**Results:**

The described model is applied to the City of Graz, a typical European city with about 320,000 inhabitants. Calculated results correspond well with reality.

**Conclusions:**

The introduced traffic model, which uses mobility data instead of origin–destination data as input, was successfully applied and offers unique advantages compared to traditional models: Mobility behavior data are valid for different systems, while origin–destination data are very specific to the region in question and more difficult to obtain. In addition, different scenarios (increased population, more use of public transport, etc.) can be evaluated and compared quickly.

## Background

Simulating and understanding (urban) traffic is an important challenge related to many different scientific fields like urban planning, transportation planning, and sustainability. The goals of traffic simulations can be manifold and include gaining insight into traffic-related noise pollution [1], finding ways to reduce emissions [2], or analyzing the effects of policies [3]. To reach these goals, many different approaches to traffic simulations exist (see, for example, [4] for a detailed overview).

In general, traffic models can be divided into two categories, namely bottom–up and top–down approaches. Bottom–up approaches start from individual vehicles and simulate their behavior to find aggregated macro-scale results. Most bottom–up approaches are micro-scale agent-based car-following models; prominent examples are the open source projects MatSIM [5] and SUMO [6] or the commercially available software VISSIM [7]. The downside of such detailed bottom–up approaches is that they need a lot of input data, in some cases complete origin–destination matrices, i.e., the complete information what vehicle drives from which starting point to which destination. In addition, their computation time is relatively high, so that calculation in real time may only be possible for small systems. A completely different approach is used for top–down models. Their starting point is the macro-scale, i.e., statistical information about traffic flow or other macroscopic properties. Such statistical models can, for example, be used to calculate emissions [8] very efficiently.

Both bottom–up and top–down approaches can utilize networks or more specifically spatial networks [9], in the simplest case to represent the road network, but also more sophisticated network methods can be incorporated in a traffic model [10], to focus on the network itself, rather than the vehicles using the road network, which can significantly speed up computation time [11].

In this study, we use a hybrid approach [12], combining features of both bottom–up and top–down methods. One of the biggest challenges in traffic simulation is to obtain realistic origin–destination data. This can be done by combining traffic count with survey data [13] or by utilizing information from a mobile-phone network [14]. In this study, we use survey data about the mobility behavior of citizens to generate realistic origin–destination data within the model. This approach has many advantages; for example, it is straightforward to evaluate various scenarios, like a certain change in mobility behavior (more use of public transport, an increased popularity of online shopping, and telecommuting) as well as juridical changes, changes in the infrastructure, or technological changes, all within the same framework. This makes the model especially powerful and versatile for the evaluation of the impact of various policies. The starting point of this study is the idea presented in [15], but goes beyond this by investigating traffic jam avoidance behavior and developing a technique to quantitatively evaluate the validity of the road usage data produced in a network-based traffic model. As a case study for evaluation we choose the City of Graz, an Austrian city with about 320,000 people, because it is a typical European city of this size.

This paper is organized as follows: "Methods" section describes the model development and gives details on the process used for evaluation. "Results" section presents the obtained results, primarily focusing on the question whether realistic road usage data could be obtained. "Discussion" section concludes with limitations and possible expansions of the presented model.

## Methods

The aim of the model presented here is to generate realistic road usage data and resulting congestion information using a network approach utilizing statistical data about mobility behavior. This process is split into six steps. First, a network representation of the investigated area is generated. In a second step, internode relations like effective distances and shortest paths are calculated. To generate a pool of realistic trips, a mobility behavior survey [16] is used. From this pool of trips, realistic road usage data are obtained in an agent-based process. Traffic jam avoidance effects are then included via an iterative method. Finally, obtained results are evaluated quantitatively.

### Generating a road network representation

**Fig. 1:**
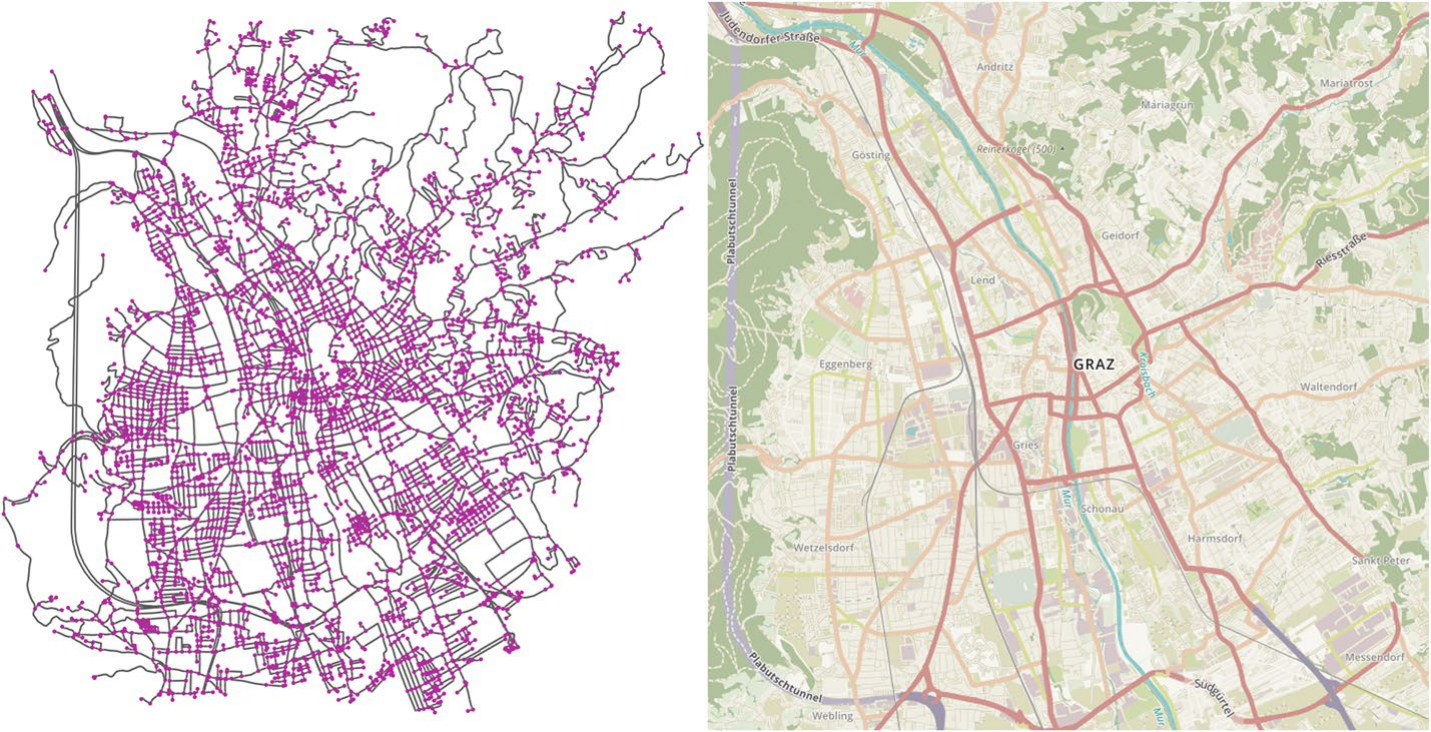
Network representation of the investigated infrastructure compared to the original map data. The left panel shows a network representation of the examined city, where the gray edges are the road segments that are connected by the purple intersections. The right panel is the map of the city from the OpenStreetMap website

To obtain a network representation of the investigated area, we use map data obtained from OpenStreetMap [17]. Such data are available for most of the world in varying degrees of detail and can include not only the geographical position of each road section, but also additional relevant data, like the width of the road, the number of lanes, the maximally allowed speed, whether it is a one-way street or not and other important features. To convert these data to a network, the library OSMnx [18] is used. This library uses the OpenStreetMap data, identifies relevant points in each road (intersections, forks, dead ends, ... ), and uses them as nodes for the network. The edges of the network are the road sections connecting the nodes, including relevant information about the road as edge variables. That way, an accurate network representation of the investigated infrastructure can be obtained in an automated way, shown for the City of Graz in Fig. 1.

Additional information that could be interesting, like, for example, population density or the position of relevant business or industrial hubs can be included via an additional OpenStreetMap import. In this example, we matched each node to a district of Graz, to utilize population density information with district resolution. Including other information was not necessary, mainly because the investigated city features a simple structure, where important business and industrial hubs coincide with high population density, yet inclusion of such data would be straightforward.

### Calculating fastest paths

During the simulation, it is often necessary to find the shortest path between two nodes, or more precisely, to find out which nodes are within a certain distance of a starting point, using shortest paths. Note that all shortest paths are weighted with the average travel time of the involved edges, so the shortest paths are in a sense fastest paths. This operation is computationally quite time consuming, so to optimize efficiency, it is performed before the simulation itself. Specifically, we calculate the length of the shortest paths between each and every node and save the information which nodes are up to x meters away from the original node, with x binned in 100 m bins. This makes finding valid target nodes (a process explained in "Calculating road usage" section) much faster. To further increase the speed of the simulation, it would be possible to use a graphics card for shortest path calculations or to store not only the distances, but the complete paths, if enough memory is available.

### Obtaining mobility behavior from survey data

The distinctive feature of the presented model is that no origin–destination data are required as input data. This information is generated from mobility behavior, gathered in the survey “Österreich Unterwegs 2013/2014” [16]. In this survey, 18,000 respondents from Austria reported their mobility behavior on 2 separate days, leading to 36,000 days of mobility data, randomly distributed over the year. Each day contains all the trips which a respondent performed (length of the trip, used transportation method, details of the car, if a car was used, and reason for the trip) as well as information about the respondent.

In principle, it would be possible to use the full set of data, but the predictive power of the model can be improved by restricting the data to data sets that were obtained in areas that are similar to the investigated system (i.e., small town, big cities,...). In this study, where the City of Graz is investigated, we restricted the mobility data to data obtained in Graz, which truncated the data set to 1800 days of mobility data.

These data are used to generate a pool of mobility behaviors, i.e., sets of daily driving behavior patterns. Each day of mobility behavior can contain several trips and each trip is primarily classified by its length and the used method of transportation. However, these data do not contain any specific origin or destination points, so, to calculate actual traffic data, we use a multi-agent approach, presented in "Calculating road usage" section.

### Calculating road usage

To generate traffic data out of the pool of mobility behaviors, detailed in "Obtaining mobility behavior from survey data" section, we generate one agent for each citizen and position those agents according to population density. To make the simulation more realistic, we differentiate both population density and mobility behavior in different age groups, to include the effect, that not all districts of a city have the same age-distribution. Once the agents are positioned, they are assigned a random mobility behavior, appropriate for their age group, taken from [16]. This gives them specific trips for this day, that they perform one after another. Since the destination nodes are not part of the trip data, but only the length of the trip a random node in the appropriate distance needs to be selected. Since the information, which nodes are at which distance from each starting node, was already calculated (see "Calculating fastest paths" section for details), the process of finding a random appropriate node is just a computationally inexpensive selection of a random element of a list of possible targets. After origin node and destination node are determined, a calculation of the fastest path between them is performed. This path is then used by the agent, using the correct mode of transportation, as given in the trip data. The destination node then becomes the origin node for the next trip and the process is repeated until all agents performed all their trips.

For certain cities, where commuting from regions outside the simulation area is relevant, it is intuitive to include such trips. Therefore, we also include agents with a starting point outside the city. Statistical data about which region they come from and which entry node is thus most likely used to enter the city were obtained from [19]. These commuting agents are especially important when investigating traffic jams, since they are mostly active during times of peak activity.

### Including traffic jam

To investigate the occurrence of traffic jams, we first need to calculate the daily traffic capacity for each section of road. For this, we need information about the width of the road, the number of lanes and if this section of road is a one-way street or not, which is already included in the road network. Using these data, we can calculate the daily traffic capacity *C* for each section of road [20]. To obtain the hourly traffic capacity *C*_h_, special conversion factors are used [21] and *C*_h_ is then calculated from

1 $$C_{{\rm h}} = L_{{\rm eff}} \, 750 \, \frac{{\rm cars}}{{\rm hour}}$$

where $L_{\text{eff}}$ is the effective number of lanes. The starting point of finding $L_{\text{eff}}$ is the actual number of lanes, if it is known. If this number is unknown, because this information is missing in the imported map data, approximate values are derived from the width of the road: $L_{\text{eff}}$ = 2.6 for roads wider than 7.5 m, $L_{\text{eff}}$ = 2.0 for roads between 7.5 and 5.5 m, and $L_{\text{eff}}$ = 0.8 for roads narrower than 5.5 m [21]. This number is then halved for roads that are not used as one-way streets, since then only half of the lanes can be used in one direction, to find the final value for $L_{\text{eff}}$ and thus *C*_h_. To find out if the traffic flows freely or a traffic jam occurs, we compare the hourly number of vehicles *V*_h_ with the hourly traffic capacity *C*_h_:

2 $$\begin{aligned} a = \frac{V_{\text{h}}}{C_{\text{h}}} \end{aligned}.$$

The obtained load quotient *a* is then used to differentiate between different stages of congestion. *a* smaller than 0.75 signifies free-flowing traffic, *a* between 0.75 and 0.9 is a sign of constrained traffic flow and *a* larger than 0.9 can be interpreted as stop-and-go traffic [21]. That way, a first approximation to the traffic conditions of each section of road can be obtained. This approximation can be further refined by including traffic jam avoidance in an iterative process. After calculating the approximated traffic conditions, agents react to the resulting congestion, by avoiding routes that are heavily congested and using alternative routes. In the model, this is done by keeping the origin–destination pairs constant (i.e., origin nodes and destination nodes of agents do not change) and recalculating the shortest paths, now including delays caused by congestion. This leads to new traffic conditions and in turn a new congestion status for every section of road in the system. This updated congestion information can again be used to refine the traffic data by recalculating shortest paths.

However, not all drivers change their route due to congestion effects. De Palma et al. [22] found that only 30% of commuters use different routes to avoid traffic jams. Thus, the starting point of our investigation is the situation that 30% of all agents are avoiding traffic jams, while the other 70% remain on their original paths at all times. In addition to this scenario, we explore more of this parameter space, by varying the percentage of congestion avoiding agents between 0 and 50%. By comparing the simulated results to congestion forecasts based on real traffic data, we find out which parameter leads to the most realistic result.

### Quantitative evaluation using congestion data

To evaluate the obtained congestion data quantitatively, statistical travel times that include congestion effects are extracted for every road segment from the Google Maps Distance Matrix API [23]. The travel times of a typical midweek morning (between 7 and 8 a.m.) with no nearby public holidays are used as peak travel times, while travel times with negligible traffic (early hours after midnight) are used as reference points. From this, the relative time lost for each section of road is calculated by the following:

3 $$\begin{aligned} t = \frac{T-F}{F} \end{aligned},$$

with the travel time with negligible traffic *F* and the peak travel time *T*. This value is then compared to the simulated results. Because the simulation yields information about the load factor *a* of each section of road and the resulting free-flowing or stop-and-go traffic, this value must be translated to relative time lost, to be comparable to the real world data. The simplest way to make these two related properties comparable is to use normalization, so that both values are between 0 and 1. In that way, the deviation *D* is calculated as follows:

4 $$\begin{aligned} D=\frac{a}{a_{\text{max}}}-\frac{t}{t_{\text{max}}} \end{aligned},$$

with the maximal load factor $a_{\text{max}}$ and the maximal time lost $t_{\text{max}}$. To gain a single scalar that scores the accuracy of the simulated values, differences are calculated, and then, the values for each section of road are weighted with the corresponding travel time and summed up. This leads to the average deviation $D_{\text{avg}}$ of the simulation results:

5 $$\begin{aligned} D_{{{\text{avg}}}} = \frac{{\sum\nolimits_{{\text{i}}} {{\text{abs}}(D_{{\text{i}}} )\;L_{{\text{i}}} } }}{{\sum\nolimits_{{\text{i}}} {L_{{\text{i}}} } }}\end{aligned},$$

with *i* running over all edges of the network, and *L*_i_ the lengths of all sections of roads. Using this equation, it is, for example, possible to compare the validity of different parameters for including traffic avoidance strategies.

## Results

### Overall road usage

The main results of each simulation run are the origin–destination data itself (which could be used as an input for a microscopic car-following model), the trajectories of all cars, and overall road usage and resulting congestion.

**Fig. 2:**
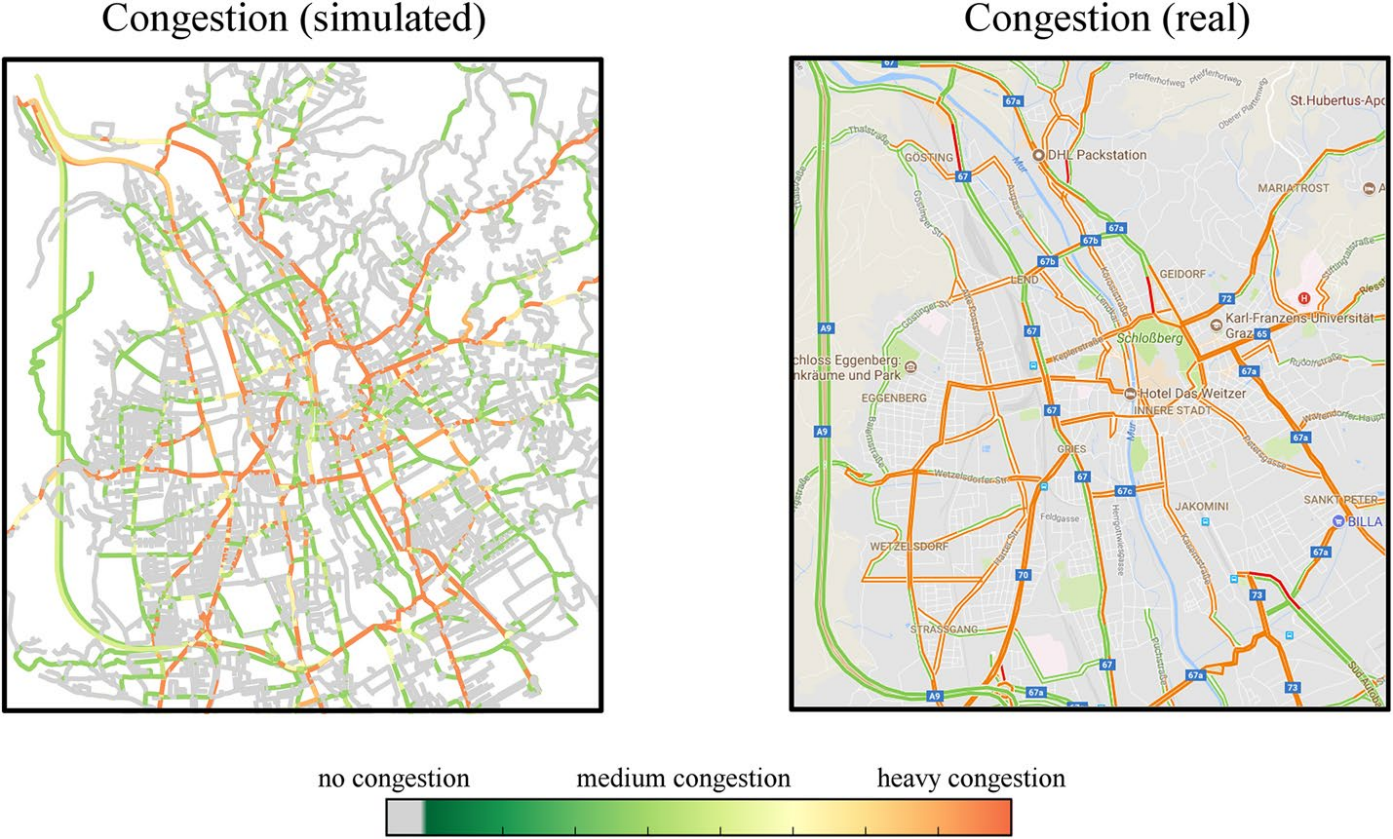
Graphical comparison between simulated congestion and real congestion. The simulated congestion of a morning rush-hour of a typical midweek day is illustrated in the left panel and the Google Maps congestion forecast of the same time frame is illustrated in the right panel

For every hour of the simulated day, the congestion is calculated as described in "Including traffic jam" section. Since the road segments are not congested most of the day and therefore not suitable for evaluation, we use the morning rush-hour between 7 and 8 a.m. for evaluation. The congestion after a first approximation of traffic without traffic jam avoidance is compared with congestion visualization from a weekday at the same time from Google Maps in Fig. 2. It shows the simulated congestion without including effects of traffic jam avoidance (left panel) compared to the actual congestion (right panel). The simulated congestion values are calculated from the load factor a, given in Eq. (2), during rush-hour. The right panel shows data extracted from Google Traffic, i.e., the amount of congestion during peak activity. Both values are in good agreement; most main travel routes are accurately depicted. This is not trivial, since no origin–destination data were used as an input for the model.

However, on closer inspection, there are some discrepancies between the simulated traffic data and the real traffic data. Mainly, the big roads are used extensively, so that a large amount of congestion occurs, while smaller, parallel roads are used only infrequently. The reason for this is that traffic jam avoidance is not included yet. Accounting for this effect significantly improves the results, as can be seen in "Quantitative evaluation and traffic jam avoidance" section.

### Quantitative evaluation and traffic jam avoidance

Including traffic jam avoidance strategies in an iterative process detailed in "Including traffic jam" section could make the obtained traffic data more realistic. However, a priori not all parameters of this technique are known. Namely, the number of iterations needed until convergence is reached is unknown, as well as the actual percentage of drivers who change their route to avoid traffic jam. To fine-tune these parameters, the method presented in "Quantitative evaluation using congestion data" section is used for the evaluation. Using this precise feedback, how well the simulation matches the reality, we can determine the unknown parameters.

**Fig. 3:**
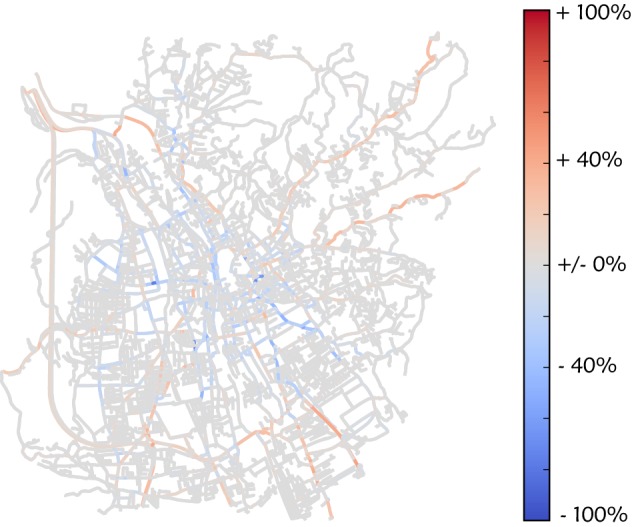
Deviation between simulation and reality without including traffic jam avoidance. The simulated congestion without traffic jam avoidance is compared with data extracted from Google Maps. The color shows the deviation relative to the maximal congestion. Red corresponds to too high simulated values, while blue means that the simulated values are too small

The results of the quantitative evaluation of the model are presented in Fig. 3. Shown is the relative error of congestion for each section of road for a simulation run without any traffic jam avoidance. Summing over all edges and weighting with the length of the section of road [see Eq. (5)] lead to an average deviation of 0.0301. While some sections of road are described very well, others show too much congestion, compared to reality, while others show too little.

**Fig. 4:**
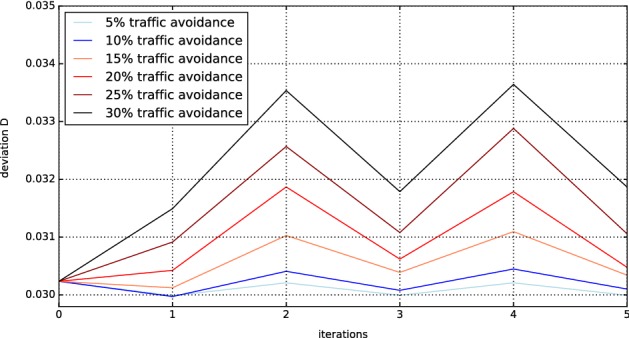
Deviation between simulated and real data with respect to traffic avoidance and number of iterations. The average deviation $D_{\text{avg}}$ for all simulation runs is compared to the reference congestion data from Google Maps

This result can be improved by the process detailed in “Including traffic jam” section. Literature suggests that 30% of commuters change their routes to avoid traffic; nevertheless, we scanned a bigger range of parameters, since especially in a city traffic avoidance behavior is not known precisely. In addition, we investigate the effect of several iterations on the validity of the simulation. This analysis is shown in Fig. 4.

**Fig. 5:**
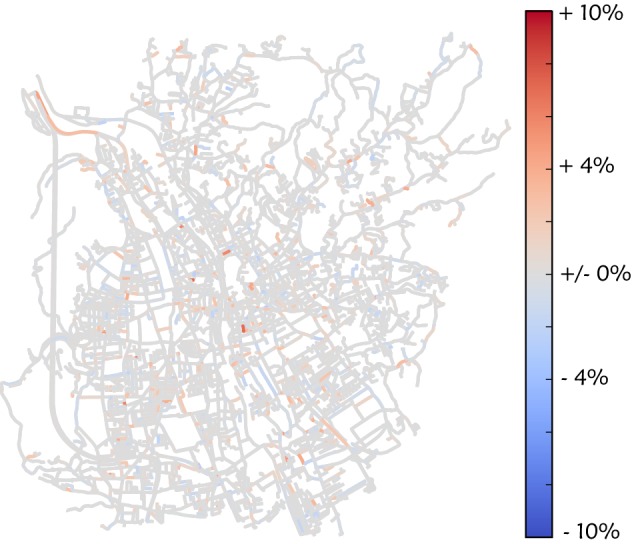
Difference in deviation due to traffic jam avoidance. Difference between one simulation run without traffic jam avoidance and the simulation run with the lowest $D_{\text{avg}}$ (10% traffic avoidance, first iteration). The color shows the change relative to the maximal congestion. Red corresponds to positive changes, while blue means negative changes

It is clearly visible that traffic avoidance chances of more than 10% cannot lead to an improvement. The best match between simulated and real data is achieved for a traffic avoidance chance of 10% after one iterative step. Note, that using a 5% traffic avoidance chance also iterations 1, 3, and 5 are in relatively good agreement, while even numbered iterations perform worse in general. The reason for this effect is the iterative nature of the traffic jam avoidance. The initial guess (0) features no traffic jam avoidance. In iteration 1, the agents avoid the heavily congested roads, making them less congested, corresponding to a more realistic picture. In iteration 2, some of the agents change back to the previously heavily congested roads, since they were less congested in iteration 1. This process repeats itself, so that the odd numbered iterations always produce more realistic results.

To visualize the positive effect of considering traffic jam avoidance, we calculate the difference in *D* between a simulation run without traffic jam avoidance and one with traffic jam avoidance (10%, first iteration). This difference is shown in Fig. 5. Although the change is relatively small, it reduces overall deviation to 0.0298, since most changes increase traffic in the inner city, which was one of the main errors (see Fig. 3).

## Discussion

It was possible to show that the presented model can quite accurately predict the road utilization of a given system without the need for origin–destination data. Precise knowledge about the mobility behavior is enough to gain satisfying results on which roads are used how often during which hour of the day. The main error is that traffic on the main roads leading into the city is overestimated, while inner city traffic is slightly underestimated. This effect has to do with the fact that agents always choose the fastest path, using the allowed speed limit to approximate travel time, while, in reality, heavily congested roads may have decreased effective speed and are, therefore, avoided by drivers. The error can be combated by including traffic jam avoidance strategies into the agents behavior.

Results showed that the best way to include traffic jam avoidance is to assume that 10% of the drivers change their route to evade congested roads. This is a lower percentage than is reported in [22]. The reason for this is that we investigated the road network of a city and that traffic jam avoidance behavior is different in an urban environment. Here, there are very few alternative paths, and even if alternative paths exist, during rush-hour, they are congested in a similar way. In addition, people as well as roads are adapted to congestion on main urban roads, so only very few drivers deviate from the prominent direct routes. One limitation of the presented model is the temporal resolution. Time is discretized in steps of 1 h. A finer discretization of time is not straightforward for the following reasons. The main advantage of the proposed model is its fast computation time, compared to the traditional car-following models. This only works, because the actual movement of all cars is simplified drastically. Actual speed and driving time are only known on averages, but there is no information about the speed, and, therefore, the current position of each car, only the exact paths are known. This simplification works quite well, as long as the assumption holds, that most of the cars are able to finish their routes within one unit of discrete time, i.e., here 1 h. This is reasonable in an urban environment. However, reducing the time steps to minutes would lead to the breakdown of this assumption. Additional information about the position of each vehicle at each minute needs to be calculated, which is much more time-consuming than the currently needed information about the path of each vehicle at each hour. Furthermore, road capacities are usually only defined in cars per day or cars per hour and it is generally advised against using road capacities for shorter time periods, since they may be inaccurate [21].

An interesting expansion would be to increase the size of the investigated road network. With the exception of calculating inter node relations, which only needs to be done once for each system, the model scales very well with the number of nodes in the system, so the actual computation time would not increase drastically. This would not only provide more information about the traffic situation outside the city, but also commuting from other cities would be included naturally, which may further improve the accuracy of the model. For even larger systems, it may be beneficial to calculate and save the shortest paths between each and every node in the system. While this process is quite memory intense and requires a lot of computation time, it needs to be done only once and, therefore, drastically speeds up computation time in the long run.

Shown here is the application of the presented model to the City of Graz, however, application to a different urban system would also be possible. Map data are available from OpenStreetMap for most regions in great detail and can be imported in the same way as presented in "Generating a road network representation" section. Mobility data were collected in Austria [16], but it is reasonable to assume that mobility behavior depends more on certain specifics of a city, rather than the country the city is in, so using Austrian mobility data of a city with similar properties and structure may yield reasonable results. Alternatively, mobility behavior data from a different source, giving details about the investigated city, could be used to further improve the descriptive power of the model. That way, the presented framework could theoretically be used to simulate an arbitrary road network in the same way as was done here for the City of Graz.

Accurately depicting the correct road usage of a given system without relying on actual origin–destination data is the basis of future investigations. Now that we were able to show that the presented model produces satisfying results with quantitative evaluation, the next step is to use this model to make predictions about various scenarios. We can make significant manipulations on the road network, or change the number of agents to account for future population development. In addition, different mobility behavior can be used to evaluate various scenarios. Since we have specific information about the reason for each trip, we can implement societal changes, like an increase in telecommuting, unemployment, or online shopping. A rudimentary analysis using an early version of the presented approach was performed in [24] and focused on emission, which are especially relevant in urban systems [25, 26]. Yet, that study lacked quantitative evaluation on a local scale and it used a simplified path finding algorithm, which completely neglects traffic jam avoidance behavior. Using the methods presented here, the analysis could be improved significantly, increasing the predictive power of the used model. Especially, for changes that affect inner city traffic and commuting behavior, an accurate depiction of congestion and congestion avoidance is paramount and including these effects in the way presented here would be a valuable expansion, making the results more realistic and accurate.
